# "Better Living with Non-memory-led Dementia": protocol for a feasibility randomised controlled trial of a web-based caregiver educational programme

**DOI:** 10.1186/s40814-023-01403-1

**Published:** 2023-10-11

**Authors:** Aida Suárez-González, Amber John, Emilie Brotherhood, Paul M. Camic, Roberta McKee-Jackson, Mel Melville, Mary Pat Sullivan, Rhiannon Tudor-Edwards, Gill Windle, Sebastian Crutch, Zoe Hoare, Joshua Stott, the Rare Dementia Support Impact team

**Affiliations:** 1 Dementia Research Centre, UCL Queen Square Institute of Neurology, UCL, London, WC1N 3BG UK; 2 Psychology and Language Sciences, University College London, London, UK; 3 Faculty of Education and Professional Studies, School of Social Work, Nipissing University, North Bay, ON Canada; 4 Centre for Health Economics and Medicines Evaluation, Bangor University, Bangor, UK; 5 Dementia Services Development Centre, School of Medical and Health Sciences, Bangor University, Bangor, UK; 6 School of Health Sciences, Bangor University, Bangor, UK

**Keywords:** Online intervention, Web-based education, Posterior cortical atrophy, Behavioural variant frontotemporal dementia, Primary progressive aphasia, Caregiving

## Abstract

**Background:**

Non-memory-led dementias such as posterior cortical atrophy (PCA), primary progressive aphasia (PPA) and behavioural variant frontotemporal dementia (bvFTD) are low prevalent and often affect individuals under the age of 65. Tailored educational and support resources for caregivers of people living with these dementia phenotypes are scarce and unevenly distributed geographically. Web-based educational programmes are emerging as promising alternatives to improve caregiver self-efficacy and well-being. Here, we present the protocol of a study aiming to assess the feasibility of a co-produced online educational programme for caregivers of people living PCA, PPA and bvFTD: the Better Living with Non-memory-led Dementia programme.

**Methods:**

A randomised controlled feasibility trial will be conducted on a sample of 30 caregivers of people living with PCA, PPA and bvFTD. Participants will be recruited among members of the support organisation Rare Dementia Support (based at UCL in the UK). The intervention group will be given access to an 8-week co-produced web-based educational programme consisting of 6 modules addressing education about PCA, PPA and bvFTD and support strategies for the person with dementia and for the caregiver. The control group will receive treatment as usual (TAU). Feasibility will be measured through feasibility of recruitment, clinical measurement tools and acceptability. Clinical measures will be used to assess preliminary efficacy and data on completion rates, missing data and variability used to decide on measures to be included in a full-scale trial.

Allocation ratio will be 2:1 (intervention:control) stratified by diagnosis. Feasibility of recruitment and acceptability will be assessed. Clinical measures will be administered at baseline and 8-week and 3-month post-randomisation. The control group will be offered access to the intervention at the completion of data collection. Participants will be unblinded, and all measures will be self-reported online.

**Discussion:**

Online-delivered educational programmes show potential for improving care competency of caregivers and may contribute to overcoming geographical inequalities in local provision of support services. This pilot study will inform a fully powered international trial to determine the effectiveness of Better Living with Non-memory-led Dementia.

**Trial registration:**

This trial has been registered prospectively on the Clinical Trials Registry on 1st September 2022, registration number NCT05525377.

**Supplementary Information:**

The online version contains supplementary material available at 10.1186/s40814-023-01403-1.

## Background

Around 48 million people worldwide [1] live with dementia, of whom 3.9 million start with symptoms before the age of 65 (young-onset dementia) [2]. Most of the people presenting with young-onset dementia and some people with later onset dementia develop non-memory-led dementias such as the atypical forms of Alzheimer’s disease (AD) [3, 4] or frontotemporal dementia (FTD) [5]. Atypical non-memory presentations of AD involve visuospatial and language dysfunction as the main clinical manifestations at onset and, more rarely, executive or motor dysfunction [6–8]. The two most common atypical presentations of AD are posterior cortical atrophy (PCA) [9, 10], characterised by progressive deterioration of visuospatial and other posterior functions, and the logopenic variant of primary progressive aphasia (lvPPA) [11], characterised by a pattern of progressive language deterioration with impaired repetition and phonologic errors. FTD is an umbrella term that encompasses a group of clinical syndromes including the behavioural variant (bvFTD) [12] presenting with behavioural (e.g. disinhibition, apathy) and cognitive symptoms (typically executive and social cognition dysfunction), the non-fluent variant of primary progressive aphasia (nfPPA) [11] where individuals affected show non-fluent speech and agrammatism and the semantic variant of PPA (svPPA) [11] characterised by fluent speech in the context of semantic knowledge breakdown.

### Online interventions for caregivers

Low caregiving competency is associated with lower quality of life in the person with dementia [13] and with the likelihood of being admitted to a long-term care facility [14]. A decreased sense of competency is also associated with feelings of hopelessness and lower mood in caregivers [15]. Despite the proven benefits of educational programmes and skill training for caregivers, families of people with non-memory-led dementias encounter fewer opportunities to receive this type of support. This is a significant gap in care considering that many people with young-onset non-memory-led dementia are in their 50s or early 60s, which carries additional challenges about employment, financial stability and childcare responsibilities [16–18]. Finding suitable information and resources is less likely due to the lower prevalence of these phenotypes, their consequent geographical spread and their atypical symptoms [19, 20]. Caregiver’s demands for more phenotype-specific support suggest that tailored provision of education and training is a gap in the provision of care in these types of dementia.

A previous clinical trial testing, a web-based blended care self-management programme [21], showed improvements in self-efficacy, mastery and quality of life in caregivers of people with dementia. Caregivers enrolled in this intervention receive personal online coaching from a trained healthcare professional, whilst they follow four self-chosen thematic modules including psychoeducation. Coaches spend around 6 h during 8 weeks supervising caregivers using the programme. This programme has been subsequently adapted to support caregivers of people with young onset and frontotemporal dementia [22–24]. The effectiveness of these modified versions has not been tested yet in a randomised clinical trial (RCT). Moreover, web-based psychoeducational programmes that require trained healthcare professionals to be delivered (as it is the case with the aforementioned) are costly to implement and sustain.

Guided by previous research in the field, and in line with stage 1 of Medical Research Council (MRC) complex intervention development and evaluation guidance [25], a novel-manualised web-based caregiver educational programme (called Better Living with Non-memory-led Dementia) was developed alongside people living with PCA, PPA and bvFTD and their family caregivers through an iterative and collaborative process (described in the methods section). This manuscript focusses on the study protocol for an RCT to test the feasibility of this programme as recommended in stage 2 of the MRC guidelines [25, 26].

## Aims and objectives

The aim of this study is to test the feasibility of the Better Living with Non-memory-led Dementia programme. The objectives are as follows:

- Primary: To assess feasibility of recruitment, feasibility of measurement tools and acceptability (prospective, concurrent and retrospective).
- Secondary: To assess the preliminary efficacy, understood as directionality on the clinical measures (that can inform optimal outcomes measure in a full trial).

## Methods

This protocol has been registered in ClinicalTrials.org (NCT05525377) and follows the SPIRIT reporting guidelines [27] (Chan et al., 2013) adapted with supplemented and replaced sections borrowed from the CONSORT extension for pilot trials, as recommended by Lancaster and Thabane [28]. The patient and public involvement (PPI) component of this study is reported following GRIPP2-SF [29].

### Study design

**Fig. 1:**
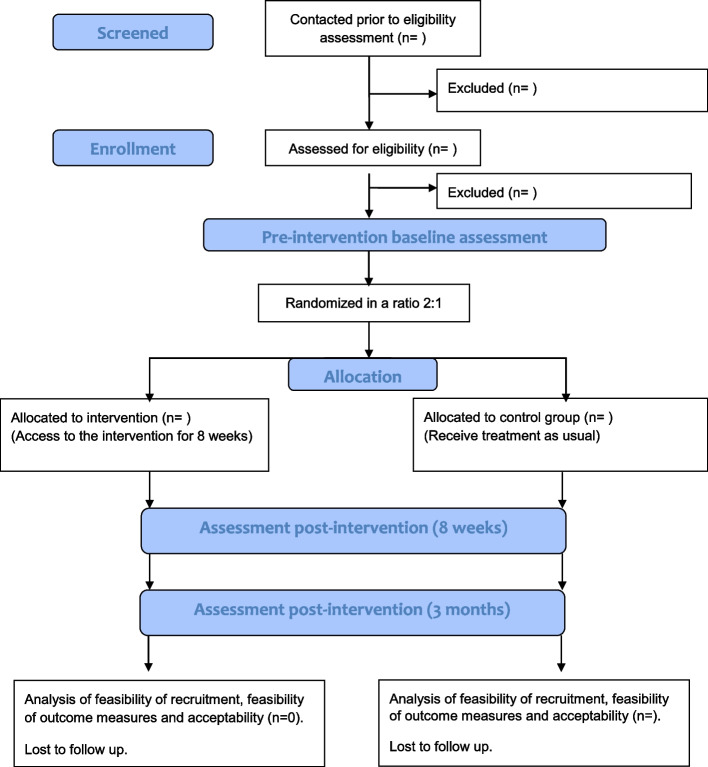
Participant flow chart of the Better Living with Non-memory-led Dementia feasibility trial

This is the protocol for a pilot randomised wait-list controlled feasibility trial followed by a qualitative evaluation (mixed-method study). Participants randomly allocated to the intervention group will receive access to the educational programme for 8 weeks. Those in the wait-list control group will receive treatment as usual (i.e. pointed to Rare Dementia Support website (https://www.raredementiasupport.org/)) and access to the programme at the end of data collection. The wait-list aimed to decrease attrition and also meet ethical demands (e.g. giving caregivers access to tailored educational resources that may potentially have a beneficial effect). Outcome measures will be collected at baseline, 8-week and 3-month post-randomisation. Semi-structured interviews will be conducted after completion of the 3-month follow-up baseline measures as part of a process evaluation. The study participant flow chart is shown in Fig. 1.

### Participants and research setting

People identifying themselves as caregivers of someone with PCA, PPA or bvFTD will be recruited from the Rare Dementia Support Impact project (RDS Impact) [30] and the wider Rare Dementia Support service (RDS) based in the UK. See Brotherhood et al. [30] for details on the ethical procedures and consent.

Inclusion criteria are as follows:

- Adults (18+) who self-identify as an unpaid caregiver (e.g. partners, children, friends) of someone with a diagnosis of PPA, PCA or bvFTD.
- The person with dementia is not living in a full-time care facility.
- The care recipient must have a confirmed diagnosis of one of these types of dementia through self-report of the caregiver (to reflect the ‘real-world’ application of the intervention).

Exclusion criteria are as follows:

- Poor comprehension of written English.
- No access to the Internet.

### Power calculation

As this is a feasibility study, a formal power calculation is not required; however, the sample size does require justification. This study was initially intended to develop a tool for carers of people with bvFTD only. However, during the interviews with stakeholders, became apparent the need and opportunity to develop two more tools, to cover PPA and PCA (details of sample calculation for bvFTD only can be found in Brotherhood et al., [31]. Originally, we aimed to recruit 135 participants comprising 45 caregivers from each of the three diagnostic groups that the intervention is designed for (PCA, PPA, bvFTD) with a 30:15 intervention control split in a 2:1 randomisation. This would have provided both a precise estimation of the overall retention rate but also within diagnostic group. Reviewing this target once recruitment was underway, we acknowledged that this would be a large feasibility study, and with a rarer disease, it would require recruiting a large proportion of the available population. Therefore, we revised the target sample down to 30 participants (randomised 20:10 in favour of the intervention); this would allow an understanding of the trial processes, allow for adaptation and refinement of the intervention and understand acceptability and fidelity within the population. The confidence intervals on our estimates will be wider, but this is an acceptable concession given the sparsity of the potential population to recruit. We will achieve a 95% confidence interval of ±18.7% around our expected value of 76% of those completing the intervention and pre-post measures whilst viewing 70+% sessions. In terms of retention to the study, we will achieve a 95% confidence interval of ±15.5% around our expected value of 75%.

#### Progression criteria

Progression criteria for the trial will be considered in a holistic way with consideration given to possible refinements and adaptations that can be made to the intervention and the trial processes to mitigate any barriers encountered. Whilst we can set quantitative thresholds indicating success on elements of the feasibility trial, these will be taken into consideration with the context of the conditions under which these were obtained, in addition to qualitative information collected. Non-progression will only be considered on the basis as to whether any of the conditions are unlikely to be resolved. Quantitative criteria will be fixed on recruitment and retention, whilst trial processes, suitability of proposed outcome measures, acceptability and fidelity will be assessed by a qualitative approach understanding the context of these. Successful recruitment will be denoted as achieving greater than 75% of proposed sample; less than 25% recruited of the proposed sample will be defined as unsuccessful and unlikely to be remedied. Successful retention will be denoted as greater than 70% of the recruited sample with unsuccessful retention denoted as less than 20% of the recruited sample.

### Recruitment

Participants will be recruited from two sources:

1. Caregivers who have already consented to be involved in the wider RDS Impact study [30]
2. Caregivers who contact us following various communications about the study (e.g. via the RDS service newsletter and social media, discussions between RDS members and RDS workers).

The consent procedure is as follows: (1) participants should first consent to participate in the wider RDS Impact study [30] of which this educational programme is a sub study, (2) participants will be sent a link to a Qualtrics page (Qualtrics, Provo, UT), at which point they will access screening questions as to their suitability for inclusion, (3) if they are screened as eligible, they will be directed to an online opt-in consent form that they should complete before given access to the survey collecting demographics and baseline measures, and (4) if they are screened as ineligible, they will be directed to a page thanking them for their interest in the study and giving them email contacts for the Rare Dementia Support service in case they want to reach out for support. Both consent forms can be found in Appendix I. Using Qualtrics display logic, those who do not consent will automatically receive a message thanking them for considering involvement in the study and asking them why they chose not to participate (being clear that they do not have to answer this if they do not want to).

We will recruit from each diagnostic group as screened. Up to fifteen semi-structured interviews will explore the experience during the study of a subgroup of participants. Individuals will be purposively sampled (by diagnostic subtype and allocation group) and approached via email asking if they want to participate. Interviews will be conducted post follow-up via Zoom.

### Randomisation

Randomisation will be provided via secure online platform hosted by NWORTH CTU, Bangor University. The randomisation procedure will be carried out by the project manager of the study (E. B.). Once consent and baseline measures have been completed, the participant can be entered into the randomisation system. Randomisation will be stratified by diagnosis (bvFTD, PPA, PCA). A dynamic adaptive randomisation algorithm will be used to maintain the allocation ratio of 2:1 in favour of the intervention and the balance within stratification variables. We intend to use an unequal allocation ratio during this feasibility study due to there being more uncertainties about the interventional arm than the control arm. Development of the intervention and adjustment to the different diagnosis groups will benefit from providing more data from the feasibility study on the intervention arm specifically. The recruiting researcher will not be aware of allocations made; randomisation will be completed by a researcher independent of the other trial processes. Given the nature of this trial, participants are not blinded to group allocation. Researchers are not blinded to group allocation either, but this does not bias measure collection since measures are self-reported online. Due to the use of unequal allocation in this stage of the evaluation, it is not possible to keep analysts blind — however, given that no inferential statistics will be generated, the risk for introduction of bias at this level is minimal. The trial will be subject to internal audit and monitoring from within NWORTH CTU. Additional regular biweekly meetings of the researchers will be conducted to monitor progress and discuss any arising issues. The whole trial will be overseen by the wider research group.

### Intervention

The intervention group will receive the Better Living with Non-memory-led Dementia programme, an 8-week duration 6-module educational programme covering the following topics: (1) welcome to the programme and what to expect from it, (2) understanding the disease, (3) how to provide better support for the person with dementia, (4) how to look after the caregiver’s own mental health, (5) where to find additional sources of support and (6) an introduction to the value of support groups. At the end of modules 2, 3 and 4, participants will be asked to complete a real-life task to put in practice skills learned in the specific module (e.g. approaching a friend and explaining the disease in lay terms). After that, every participant will be encouraged to reflect on that experience and share it with a programme facilitator via email. The facilitator will be a member of the team, with no clinical training, who will interact with the participant via email sticking to principles of active engagement (a script has been developed on this purpose based on principles of active listening adapted to written communication (see Appendix II). The facilitator will not act as an advisor as to the use of therapeutic techniques. All course modules will be printable using the PDF download button on the course’s page. An online version of the template for interventions description and replication (TIDIER) [32] checklist for this intervention can be found at tidierguide.org/#/gen/P2-xFB4ZH.

#### Intervention development

This educational programme was developed following the MRC guidance for development of complex interventions [25, 26, 33] comprising the following stages:

##### Stage 1: Identifying existing evidence and interventions

The initial idea about the development of this educational programme was prompted by the experience of the research team (consisting of academics, clinicians and support workers specialised in non-memory led dementias) interacting with people with PCA, PPA and bvFTD and their families over the years. Rare Dementia Support, a specialised national support service for these types of dementia, is run by our group at UCL. In this first stage of the development, the authors conducted a revision of the most up-to-date literature on the broad topic of provision of online support for caregivers of people with dementia, extracting information about relevant variables such as follows: intervention content, theories used for logic models, information about skills taught to caregivers in previous interventions, resources used to support learning, outcome measures, barriers and facilitators to caregiver engagement, details about how other blended interventions have been articulated, whether they work and if they are sustainable and have achieved successful implementation. A second step involved the revision of existing tools (both for online and face-to-face delivery). Since most online interventions fail to reach the implementation phase [34], it is not surprising that we found far more papers describing the efficacy of the tools than tools themselves which were available for the research team to test. The quality of the studies conducted also varied in quality. For the purpose to inform our intervention, we scrutinised a selection of studies that met the following criteria: (1) had a comparator group and (2) had a N above 20 in each arm (either RCT or pilot studies) [21, 35–49]. In a third step, we drew on the experience of colleagues leading the development of or administering online interventions. We therefore held a series of meeting during the evidence gathering phase. We met with research colleagues involved in the development of Communication Bridge [50], Partners in Balance [22] and Rhapsody [24], three tools recently developed or adapted for people with young onset and non-memory led dementias. Meetings with NHS clinicians working in memory services and IAPT services and experience in the use of digital tools were also held. Multiple websites offering online information, training, therapy and support were reviewed to inform web design, participant flow and user experience (e.g. https://www.dementiacarecentral.com, https://www.beatingtheblues.co.uk, https://www.partnerinbalans.nl/statics/de/).

##### Stage 2: Identifying theory

This educational programme is informed by theories of self-efficacy [51], behaviour change [52], coping theory [53] and social learning theory [51]. Self-efficacy is the person’s belief that one can perform competently and capably in given situation [51]. Caregivers with low self-efficacy beliefs tend to focus on their personal inefficiencies, the difficulty of the task and the negative consequences of failure. Self-efficacy is an important component of behaviour change. The COM-B model of behaviour change [52] argues that behaviour comes from an interaction of three factors: capability to perform the behaviour, opportunity and motivation to carry out the behaviour. Interventions for caregivers need to alter one or more of these three elements to achieve change. To select the most suitable behaviour change techniques (BCTs) for our intervention, we used the Theory and Techniques Tool [54] (https://theoryandtechniquetool.humanbehaviourchange.org/tool). This tool also provides potential links between the BCTs and mechanisms of action, which informed the format and content of the manuals of our programme. Coping refers to the strategies that individual use to manage stress [53], and these strategies may in turn affect the overall well-being of caregivers [55]. In the context of caregivers of people with dementia, coping strategies and social support can act as potential mediators between caregiver stressors and health outcomes [56]. Lastly, social learning theory [51] posits that people learn from each other from observing, modelling and imitating their behaviour. This latter theoretical framework influenced the format of the resources used in this educational programme (e.g. abundant vignettes and case studies, links to recordings of support group meetings and interviews with caregivers).

##### Stage 3: Modelling process and outcomes

**Fig. 2:**
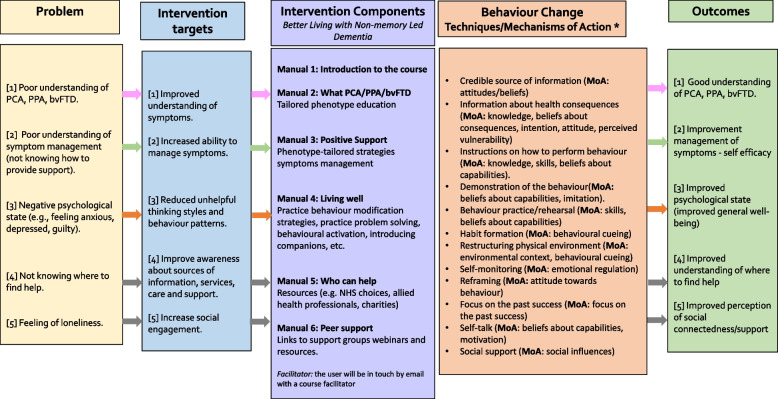
Logic model for the Better Living with Non-memory-led Dementia programme

Information from stages 1 and 2 was pulled together to develop a logic model (shown in Fig. 2). It was decided that the educational programme would consist of 6 modules or manuals following the structure of previous interventions for caregivers [22–24]. The broad content of the educational programme manuals was decided based on the intervention targets and the co-production work with people with lived experience described in the section below. The specific way to bring that content to life was shaped by the behaviour change techniques and mechanisms of action that were deemed to drive effective change and are listed in Fig. 2. According to our logic model, the outcomes of the educational programme are expected to include changes in caregiver self-efficacy, relationship with the care recipient, psychological status, quality of life and health and social health. The group of people with lived experience who co-produced the programme manuals also contributed to inform the clinical outcome measures used in the pilot feasibility trial.

##### Development of the manuals: coproduction with experts by experience

The 6 modules/manuals of Better Living with Non-memory-led Dementia have been co-produced with a group of experts by experience (EbE) (people with dementia and/or their relatives) through an iterative process with investigator ASG. Members of the organisation Rare Dementia Support were invited to join the co-production group. Distribution of a call for experts by experience flyers was used for this purpose (these flyers were co-produced with a couple of EbE and can be found in Supplementary material). Twenty-one EbE joined the co-production group between end of 2019 and 2021. Due to the Covid-19 pandemic, most meetings took place over videoconference and, exceptionally, phone or face to face (17 videoconferences, 1 phone call and 1 face-to-face meeting). There was also extensive correspondence by email between the EbE group and ASG. The subgroup of EbE working on the manuals for bvFTD was entirely composed by relatives of people with bvFTD, and meetings took place as a group. This was due to the difficulty to engage people with bvFTD in the process given the behavioural features and lack of insight characterising the condition. In the groups working on the PCA and PPA manuals, there was representation of people living with the condition. To support the participation of the person with dementia, these meetings took place one to one between ASG and the person living with dementia and their relatives. Briefly, the procedure for the meetings involved (1) sending supporting material before the meeting and information about how to prepare (created following easy-to-read and inclusive guidelines); (2) asking about preferred forms of communication prior to the meeting; (3) sending questions in advance to be asked during the meeting, so the person with dementia could have time to think and write down their responses if needed; and (4) sending minutes after the meetings in an accessible format for the person with dementia and their relatives to review together. This last step proved an important opportunity to correct inaccuracies and misunderstandings.

In the early stages of the development of the manuals, the work with the EbE group focused on the structure of the manuals and main messages to come across. For instance, one of our EbE with PPA wrote, ‘I would like people to understand that this is the way I speak, I have a condition, people need to be more patient’. The husband of a person with PCA said about the manuals, ‘they should be written in a positive way and convey hope’. The EbE group provided extensive advice about what symptoms to address in the programme and how, top priorities in management of symptoms, what strategies were more important to consider, they shared real situations to be include as vignettes, made suggestions about options for respite and support, identified common barriers to implement symptom management strategies and shared tips of how they had overcome them, how they managed difficult emotions (e.g. shame, guilt), deal with legal issues, relationships with others, and how they introduced companions and advice on what vocabulary to use in the manuals. The group preferred that all the material and resources from the course were also provided in a printable format (not only on the web) so people could share it with relatives and friends. The group also advised on the development of the website to host the education programme. Wireframes of the website were shared with the group either in videoconference meeting or by email, and their feedback was incorporated. In the late stages of the development process, drafts of the manuals were circulated by email for EbE to review. It was predominantly EbE who were relatives, and not people with dementia, which contributed to this last round. They sent feedback as notes in emails or direct corrections in the drafts of the manuals (either typed or handwritten). The group would also share personal resources they had found useful to understand their relative’s condition, to manage symptoms and their own well-being. Some members of the EbE group also contributed to the final proofreading.

The work of co-production took place between October 2019 and March 2022. The final draft of the manuals also received input from educational group facilitators and went through several rounds of proofreading with academic and clinical colleagues.

##### Content of the manuals

Six learning modules per phenotype (PCA, PPA, bvFTD) resulted from the co-production phase, making 18 in total. The first 3 modules are phenotype specific. The fourth one is common across phenotypes since it is focussed on caregiver wellbeing. The fifth module in each set contains a list of resources relevant across phenotypes (e.g. charities and organisations that can provide support), and the sixth one is an introduction to phenotype-specific support groups with links to three support group webinars each. Together, the 18 manuals contain 49 purpose-build illustrations, 53 vignettes and 44 links to external resources (e.g. videorecording of support meetings).

##### Accessibility

In Brotherhood et al., [31], we referred to enhancing the accessibility of the intervention for those with disability. However, during co-production, it became apparent that such measures were not necessary. The PPI group noted that there are already many freely available accessibility tools widely used by the community of people with reading and visual disabilities.

### Comparison

The control group (wait-list) will receive treatment as usual, consisting of explicit signposting to the publicly available Rare Dementia Support website and will continue with any kind of support the participants may already being receiving (e.g. psychological support, online information, support groups). They will be given access to the intervention at the end of the study.

### Procedures

The flow of events for participants in this study is as follows:

1. Participants give online consent to take part in the study.
2. Once consent is given, they receive a link to a Qualtrics survey to complete online demographic information and baseline outcome measures.
3. After the surveys are filled out, participants are randomised and notified of whether they will be given access to the intervention or to treatment as usual.
4. Participants in the intervention group receive an email with a link (unique for each participant) to access the online intervention. Clicking on this link will give them access to a bespoken online platform purposely built for this study. Participants are encouraged to go through the 6 modules comprising the programme (the 6 modules are available at the same time). They are also encouraged to engage with the programme facilitator via email, to reflect on their experience putting in practice the skills learnt in modules 2, 3 and 4. The intervention is tailored to each phenotype: participants caring for a person with PCA only access the PCA modules, etc.
5. Participants in the control group are redirected to an existing website about rare dementia. They do not receive additional support.
6. Eight weeks after randomisation and access to the programme (or redirection to the website) has been granted, participants receive a new link to Qualtrics to complete follow-up outcome measures. This is repeated 3 months after randomisation.

### Data collection, management and analysis

#### Demographics

After giving consent, participants will fill in an online survey with demographic information before start completing the baseline measures.

#### Feasibility measures

The following feasibility measures will be collected:

- Recruitment process
  Number of people agreeing to be sent information about the study.
  Proportion of participants who agree to participate.
  Proportion of eligible participants who agree to participate.
  Potential inequalities in recruitment feasibility will be assessed by comparing basic data on ethnicity age, gender and diagnosis of care recipient between Rare Dementia Support members, those who are screened as eligible for and those who consent to the study.
- Retention
  The analysis will consider the points from the CONSORT checklist for randomised pilot and feasibility trials [57] to ensure that all topics are being covered. Values for eligibility rates, recruitment rates, attrition rates and withdrawal rates will be reported using the participant flow data collected within the study. This will be evaluated overall and per group.
  Furthermore, details on reasons for ineligibility and non-recruitment will be reported within a table along with their related patient frequencies and percentages. Information on withdrawals and nonrespondents will be presented including reasons where applicable and time points during the trial.
- Feasibility of measurement tools
  Time taken to fill in questionnaires (with consideration for outlier times to be those where participants have left the questionnaire to complete at another time).
  Missing data from questionnaires (questionnaires will be implemented using the Qualtrics function that reminds participants when they have not filled in an item, but will not force a choice).
  Follow-up response rates (8-week and 3-month post-randomisation).

#### Acceptability measures

We used the definition of acceptability recently proposed by [58] to decide on the following acceptability measures:

##### a) Prospective acceptability 

- Burden (reasons for not taking part or discontinuation).
- Percentage who completed baseline measures agreeing to be randomised and reasons for not taking part in randomisation.
- Answers to qualitative questions in baseline questionnaire about how they feel about the intervention (e.g. how do you feel about taking part in this course?), burden (e.g. how much effort do you think will be involved in taking part in this study?) and ethicality (e.g. do you have any ethical concerns about taking part in the course? If yes, what would these be? (See Appendix  III).

##### b) Concurrent acceptability

- Intervention coherence and adherence
  o Task completion rate after every module.
  o Number of interactions with facilitator.

##### c) Retrospective acceptability 

It will be measured in two ways:

1. To capture views of everyone participating in the study, follow-up measures will include a brief acceptability questionnaire designed to assess opportunity costs and perceived effectiveness. The health economic questions used for the control and intervention group will differ: participants in the intervention arm will be asked questions on resource use and questions on willingness to pay, whilst those in the wait-list control arm will be asked about resource-use only. Participants in the wait-list control arm will be asked about acceptability of filling in the measures at each time point. Participants in the intervention group will be asked about acceptability of the intervention (see Appendix III).
2. A more in-depth qualitative interview focussed on the above headers (as well as mechanisms of change) will be given to a smaller number (n~15) of purposively sampled individuals.

#### Proposed clinical measures

The range of clinical outcomes encompassing self-efficacy, relationship with care recipient, psychological status, quality of life and health and social health will be assessed at baseline line, 8-week and 3-month post-randomisation. An additional section about health economics will be added to explore the feasibility of evaluating the costs of the educational programme in a larger trial. We will be using the following measures:

1. WHO 5 Well-being Index [59]
2. GAD-7 [60]
3. PHQ-9 [61]
4. De Jong Gierveld Loneliness Scale [62]
5. Lubben Social Network Scale [63]
6. Pearlin Mastery Scale [64]
7. Caregiver Self-Efficacy Scale [65]
8. Dementia Management Strategies Scale [66]
9. The quality of carer-partner relationship scale [67]
10. Health economics questions (developed by the health economic collaborators, RTE and BA) (see Appendix  III).

The recommended SPIRIT schedule for the enrolment or participants, administration of the intervention and assessment time points is shown in Table 1.

**Table 1:**
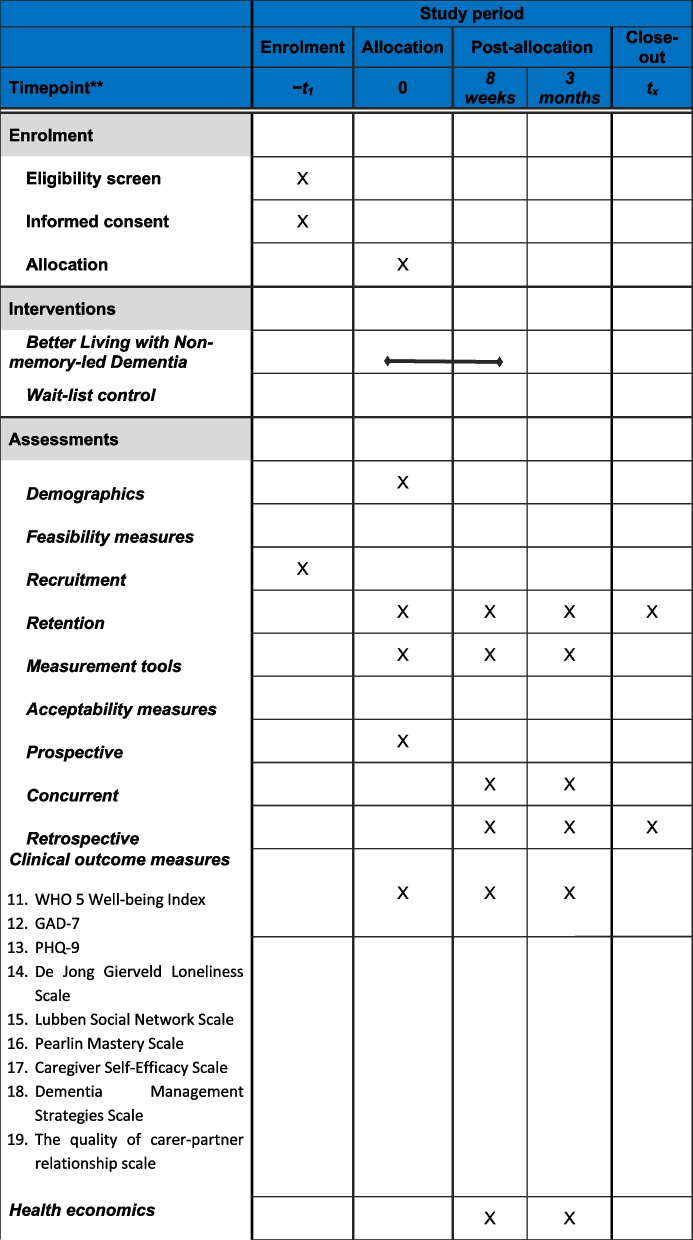
SPIRIT participant timeline with time schedule of enrolment, intervention implementation, and assessment time points

^*^Recommended content can be displayed using various schematic formats. See SPIRIT 2013 explanation and elaboration for examples from protocols

^**^List specific time points in this row

### Statistical analysis

#### Quantitative data

All analysis will be guided by the principle of intention to treat. There will be a preliminary analysis of intervention outcomes. Point estimates and 95% confidence intervals will be calculated using adjusted means from the analysis and used to estimate standard deviations and effect sizes for continuous data. Means and standard deviations of response rates for count data and proportions in each category will be provided. These will be used to confirm the sample size calculation for a definitive study. Dependent on the recruitment within each diagnosis group, we may consider presenting preliminary results within diagnosis groups. Exploratory analysis will be performed to determine the most appropriate model of analysis for a definitive RCT, including consideration of the further possible covariates and factors to be included in an analysis model. All quantitative analysis will be completed using Stata 17, SPSS v25 and R version 3.

#### Qualitative data

Semi-structured online interviews for collecting data on participant experience and programme usability will be recorded via Zoom and transcribed verbatim. Transcripts will be analysed using thematic analysis [68]. Members of the research team with experience in thematic analysis will be involved in the qualitative analysis of data and will assess data saturation throughout the Zoom interview process.

## Discussion

This is a protocol paper for a feasibility study that will serve to inform the first large-scale RCT of an educational intervention for caregivers of people living with PCA, PPA and bvFTD. Whilst emerging evidence suggests that caregivers of people with young onset dementia and FTD can benefit from web-based psychosocial programmes, there is a dearth of digital tailored interventions for PCA, PPA and bvFTD specifically. Online provision of support in the form of educational programmes might be beneficial for improving caregiver self-efficacy and psychological and health-related clinical outcomes. However, the scarcity of RCTs yields uncertainty about whether the delivery of such programmes is effective and suitable to be rolled out at large scale. Our study will contribute to respond to these questions and to advance our understanding of digitally delivered and self-administered training programmes for carers of people with non-memory-led dementias.

### Supplementary Information

**Additional file 1: Appendix I:** Consent forms for the broad Rare Dementia Support Impact Study (RDS Impact) (A) and opt-in online consent form for sub study “Pilot feasibility study for the Better Living with Non-memory led Dementia caregiver educational programme” (B).

**Additional file 2: Appendix II:** Active Engagement Scripts for interaction with participants of Better Living with Non-memory led Dementia educational program.

**Additional file 3: Appendix III:** Extension of survey to assess prospective acceptability, retrospective acceptability and health economics.

## Data Availability

De-identified project data will be available via a data depository at grant end (e.g. UK Data Service) in accordance with ethical approvals and grant stipulations.
