# Supplementary material for: "Better Living with Non-memory-led Dementia": protocol for a feasibility randomised controlled trial of a web-based caregiver educational programme
Source: Pilot Feasibility Stud. 2023 Oct 11;9:172. doi: 10.1186/s40814-023-01403-1 (PMC10566043; doi:10.1186/s40814-023-01403-1)
Supplement: Supplementary file 1 — Additional file 1: Appendix I: Consent forms for the broad Rare Dementia Support Impact Study (RDS Impact) (A) and opt-in online consent form for sub study “Pilot feasibility study for the Better Living with Non-memory led Dementia caregiver educational programme” (B). [file 40814_2023_1403_MOESM1_ESM.docx]

**Appendix II.** Consent forms for the broad Rare Dementia Support Impact Study (RDS Impact) **(A)** and opt-in online consent form for sub study “Pilot feasibility study for the Better Living with Non-memory led Dementia caregiver educational programme” **(B)**

**_________________________________________________________________**

**CONSENT FORM A) RARE DEMENTIA SUPPORT IMPACT STUDY**

This study has been approved by the UCL Research Ethics Committee: Project ID number: 8545/004

Please complete this form with a member of the research team after you have read the Information Sheet and/or listened to an explanation about the research.

Department: University College London (UCL) Queen Square Institute of Neurology

Name and Contact Detailsfor the research team:

- [research@raredementiasupport.org](mailto:research@raredementiasupport.org)
- Principal Researcher: Prof. Sebastian Crutch: s.crutch@ucl.ac.uk

Name and Contact Details of the UCL Data Protection Officer: Alexandra Potts: data-protection@ucl.ac.uk

Thank you for considering taking part in this research. The person organising the research must explain the project to you before you agree to take part. If you have any questions arising from the Information Sheet or explanation already given to you, please ask the researcher before you decide whether to join in. You will be given a copy of this Consent Form to keep and refer to at any time.

Please read through each item below. If you are happy with what you have read, either:

● For the purpose of the recording, please say ‘Yes’ each time after the researcher has given the item number or: Initial each box below

*Saying ‘yes’ or adding your initials next to each item indicates your* consent to this element of the study. It will be assumed that where you say ‘no’/do not initial a box, it means that you DO NOT consent to that part of the study .If you do not give consent for particular elements, it may mean that you will not be able to take part.

1. About the study:

1.1 I confirm that I have read and understood the Information Sheet (Version 6-C, 08-02-22) for the above study. I have had an opportunity to consider the information and understand what will be expected of me. I have also had the opportunity to ask questions which have been answered to my satisfaction.

1.2. I consent to an audiovisual recording being made of me as part of this study.

1.3. I understand that I will be able to withdraw from the study at any time, and that my data in the study up to that point will be retained by the research team.

2. Your study preferences

2.1. *I would like to take part in (please indicate one or more of the following)*

| Individual interviews/discussions |
| --- |
| Joint interviews/discussions with a relative/friend present |
| Group discussions with other research participants present |
| Testing online programmes |
| Completing questionnaires and scales |
| Taking part in research poetry |

3. About your data we will collect from this research

3.1. I understand that all personal information will remain confidential within the research team and that all efforts will be made to ensure I cannot be identified.

3.2 I understand that my personal data will be held securely by UCL, and analysed by the RDS Impact study team members from: UCL, Bangor and Nipissing University (Canada) as part of this research collaboration.

4. Sharing your data

4.1 I understand and agree that comments and contributions I may make during the research process may be presented anonymously in a publication or educational setting.

4.2 I understand that information about me and my participation in this research may be shared with other institutions and collaborators, and that this information will not contain any detail that identifies me.

4.3. I understand that, where I have taken part or will take part in other research at the Dementia Research Centre, UCL, my research data may be shared with these research teams where relevant

4.4 When the study finishes, I consent to the data I provide to be archived on secure servers at UCL, and an anonymous version to be uploaded to a data repository for other health researchers to use in the future.

4.5. I understand that my information may be reviewed by individuals from the University (e.g. UCL and our funders NIHR/ESRC) for monitoring and audit purposes.

4.6. I understand that my data will not be made directly available to any commercial organisations for profit or onward processing beyond the purposes of this study

5. Risks and benefits to taking part:

5.1. I understand how I will be supported in the event I become distressed during the course of the research.

5.2. I understand that I will not benefit financially from this study or from any possible outcome it may result in the future.

5.3. I am aware of who I should contact if I wish to lodge a complaint

6. I agree to take part in the above study. I understand I will not receive any individual feedback or recordings from the study procedures.

Signing the document:

State or write your name here: _________________________

Signature (if written form): _________________________

Researcher name stated or written here: _________________________

Researcher signature (if written form): _________________________

_Date_ stated or written here_:_ ______________________

**CONSENT FORM B) Pilot feasibility study for the Better Living with Non-memory led Dementia caregiver educational programm.**


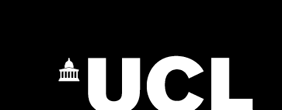


**Pilot feasibility study for the Better Living with Non-memory led Dementia caregiver educational programme - RDS Impact Study**
 
Thank you for your interest in this research. This study is part of the RDS Impact Study, ethical approval for which has been granted by the UCL Research Ethics Committee. **You can read the full information sheet for the study by clicking**[**here.**](https://uclpsych.eu.qualtrics.com/CP/File.php?F=F_bPEPdd6vEBFGLCm)

Before taking part, you will need to provide your consent to take part in the study by answering the question below.

Do you confirm that you have read and understood the information provided about this study and are happy to complete this online questionnaire?


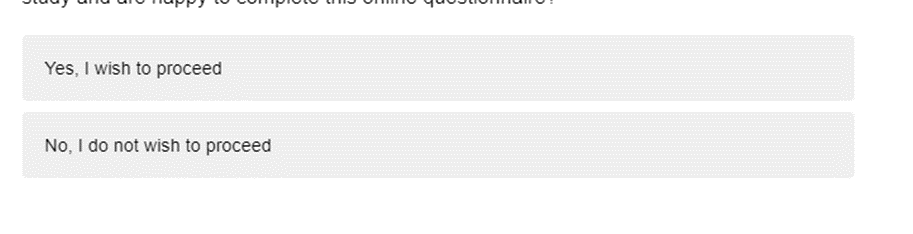


* IF PARTICIPANT SELECTS YES, THEY CONTINUE ON TO THE STUDY. IF THEY SELECT NO, THEY SEE THE FOLLOWING

Thank you for your interest in this study. You have not provided consent to participate. If this was a mistake, please select the option below to return to the previous screen.

Take me to the previous screen

You may be eligible to take part in other parts of RDS Impact, and if your responses reflect this, a member of the research team will reach out to you directly’

Below is the email contact for the RDS service in case you want to reach out for support.

[**contact@raredementiasupport.org**](mailto:contact@raredementiasupport.org)

**OPTIONAL:**What is the reason you chose not to participate in this study?
