# Supplementary material for: "Better Living with Non-memory-led Dementia": protocol for a feasibility randomised controlled trial of a web-based caregiver educational programme
Source: Pilot Feasibility Stud. 2023 Oct 11;9:172. doi: 10.1186/s40814-023-01403-1 (PMC10566043; doi:10.1186/s40814-023-01403-1)
Supplement: Supplementary file 2 — Additional file 2: Appendix II: Active Engagement Scripts for interaction with participants of Better Living with Non-memory led Dementia educational program. [file 40814_2023_1403_MOESM2_ESM.docx]

**Active Engagement Scripts for interaction with participants of Better Living with Non-memory led Dementia educational program.**

**___________________________________________________**

Active Listening is a Communication Skill. It has three steps:

1. Show that you are listening.
2. Encourage sharing and,
3. Show that you are making the effort to understand.

This usually requires verbal and non-verbal cues. However, we won´t be able to use non-verbal cues in this case because interaction with participants will take place entirely by email. That’s why we’ll refer to our way of interacting as “active engagement” instead of “listening”.

We will be using active listening to 1) encourage people to put in practice a new skill AND 2) make sure participants feel that there are real people behind the course, and we care about their experience (this has emerged as a relevant component of blended interventions in previous studies).

We won´t be using the email correspondence with participants to 1) provide light-touch therapy or advice, 2) discuss a topic different from putting skills into practice.

___________________________________________________________________________

We will use two versions of the same script to guide the interaction with the participants in the Conversation box:

**Version A: Participants share their experience.**

1. *Message from participant comes in.*
2. *Facilitator responds:*

- *show that you are* ***listening*** *and* ***understanding*** *(address participants by their names, respond to what they say, let them know you are looking forward to see how they build skills along the way)*
- *provide* ***positive reinforcement*** *(praise their achievement) and*
- *encourage the participant to* ***move*** *to the next course chapter and* ***get in touch again*** *when the next module is over).*

*Close interaction here.*

**Version B: Participants share their experience AND they have encountered problems.**

1. *Message from participant comes in.*
2. *Facilitator responds:*

- *show that you are* ***listening*** *and* ***understanding,***
- *provide* ***positive reinforcement*** *(praise what they have done well)*
- ***encourage sharing more information*** *to understand what went wrong.*

1. *Message 2 from participant comes in.*
2. *Facilitator response 2:*

- *show that you are* ***listening*** *and* ***understanding****,*
- *provide* ***positive reinforcement*** *(praise what they have done well)*
- *encourage the participant to* ***move*** *to the next course chapter and* ***get in touch again*** *when the next module is over).*

*Close interaction here.*

**Toolkit to show that you are listening and understanding and to encourage sharing.**

**Show that you are listening**:

- “I see”
- “I understand”

Use paraphrasing:

This is a helpful paraphrasing tool if you run out of brain power :): <https://quillbot.com>

You can use paraphrasing to respond in useful ways to people who express discouragement, e.g.: “There is nothing I can do to improve this situation” ⇒ “It seems you feel you can´t find a way to improve the situation at this moment”. What you are doing here is paraphrasing AND reframing in a subtle way, without challenging the person.

**Show you are making the effort to understand:**

Summarise and ask for clarification if there is something you didn´t understand:

- “What I´m hearing is … “
- “Sounds like you are saying …”
- “It looks like that made you feel …”
- “I´m not sure I understand ….”

Don´t counter argument

**Encourage sharing**:

Questions:

- “Can you tell me more about ….?”
- “What are your thoughts about …?”
- “How did you feel when …?”
