# Supplementary material for: "Better Living with Non-memory-led Dementia": protocol for a feasibility randomised controlled trial of a web-based caregiver educational programme
Source: Pilot Feasibility Stud. 2023 Oct 11;9:172. doi: 10.1186/s40814-023-01403-1 (PMC10566043; doi:10.1186/s40814-023-01403-1)
Supplement: Supplementary file 3 — Additional file 3: Appendix III: Extension of survey to assess prospective acceptability, retrospective acceptability and health economics. [file 40814_2023_1403_MOESM3_ESM.docx]

**Appendix III –** Extension of survey to assess prospective acceptability, retrospective acceptability and health economics.

1. **Prospective acceptability**
2. How do you feel about taking part in this study?
3. How much time do you think will be involved in taking part in this study?
4. How much effort do you think will be involved in taking part in this study?
5. Do you have any ethical concerns about taking part in this course?
6. What ethical concerns do you have?
7. **Retrospective acceptability - asked at follow up only**
8. On a scale of 1 (didn´t understand at all) to 10 (fully understood), how much did you understand this course and how it works? Please explain your answer.
9. How confident were you that you would perform the tasks required of you through the study?
10. What did you like about the study?
11. If we were doing this study again in the future, what should we be doing differently?
12. On a scale of 1 (purpose was not achieved at all) to 10 (purpose was fully achieved) do you feel this study achieved its purpose? Please explain your answer.
13. Did you need to give up things you enjoyed, value or profit to do this study?
14. What were the things that you gave up doing this study?
15. How did you feel about taking part in this study?
16. How much time was involved in taking part in this study?
17. On a scale of 1 (no effort at all) to 10 (so much effort I had to stop the study), how much effort was required to participate? Please explain your answer
18. Did you have any ethical concerns at any point during this study?
19. What ethical concerns did you have?
20. Do you have any other comments on the study?
21. **Health economics**

*Completed only by intervention group*

1. The intervention that you are taking part in is free of charge, but if in the future the program was ever marketed as a business to keep it sustainable, would you be willing to pay a price to be involved?
2. How much do you think this service should cost per year?
3. Do you think this should be means tested in some way? (i.e. should be tested to determine whether an individual or family is eligible for funding to pay for the programme?
4. What is your household income?

*Completed by control and intervention group*

1. Have you talked to a health care professional in the last 8 weeks about anything in relation to your caring role?
2. What type of healthcare professional did you speak to?
3. How many times did you speak with this healthcare professional?
